# Purinergic mechanosensory transduction and visceral pain

**DOI:** 10.1186/1744-8069-5-69

**Published:** 2009-11-30

**Authors:** Geoffrey Burnstock

**Affiliations:** 1 Autonomic Neuroscience Centre, Royal Free and University College Medical School, Rowland Hill Street, London NW3 2PF, UK

## Abstract

In this review, evidence is presented to support the hypothesis that mechanosensory transduction occurs in tubes and sacs and can initiate visceral pain. Experimental evidence for this mechanism in urinary bladder, ureter, gut, lung, uterus, tooth-pulp and tongue is reviewed. Potential therapeutic strategies are considered for the treatment of visceral pain in such conditions as renal colic, interstitial cystitis and inflammatory bowel disease by agents that interfere with mechanosensory transduction in the organs considered, including P2X_3 _and P2X_2/3 _receptor antagonists that are orally bioavailable and stable *in vivo *and agents that inhibit or enhance ATP release and breakdown.

## Introduction

**Figure 1:**
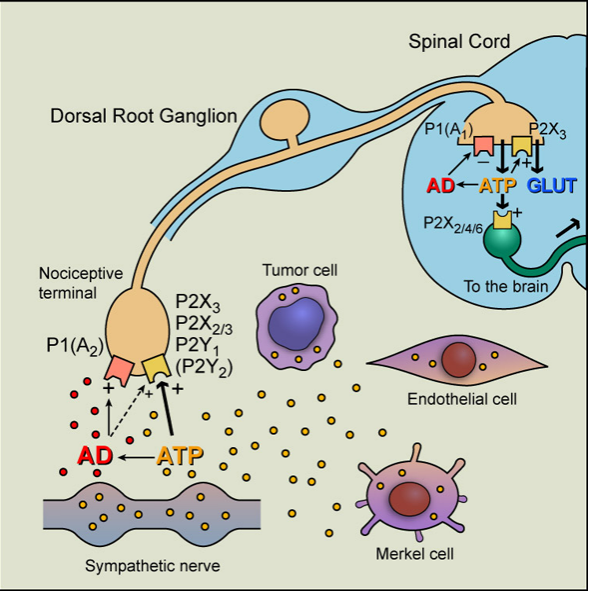
**Hypothetical schematic of the roles of purine nucleotides and nucleosides in pain pathways**. At sensory nerve terminals in the periphery, P2X_3 _and P2X_2/3 _receptors have been identified as the principal P2X purinoceptors present, although recent studies have also shown expression of P2Y_1 _and possibly P2Y_2 _receptors on a subpopulation of P2X_3 _receptor-immunopositive fibers. Other known P2X purinoceptor subtypes (1--7) are also expressed at low levels in dorsal root ganglia. Although less potent than ATP, adenosine (AD) also appears to act on sensory terminals, probably directly via P1(A_2_) purinoceptors; however, it also acts synergistically (broken black line) to potentiate P2X_2/3 _receptor activation, which also may be true for 5-hydroxytryptamine, capsaicin, and protons. At synapses in sensory pathways in the CNS, ATP appears to act postsynaptically via P2X_2_, P2X_4 _and/or P2X_6 _purinoceptor subtypes, perhaps as heteromultimers, and after breakdown to adenosine, it acts as a prejunctional inhibitor of transmission via P1(A_2_) purinoceptors. P2X_3 _receptors on the central projections of primary afferent neurons in lamina II of the dorsal horn mediate facilitation of glutamate and probably also ATP release. Sources of ATP acting on P2X_3 _and P2X_2/3 _receptors on sensory terminals include sympathetic nerves as well as endothelial, Merkel, and tumor cells. Yellow dots, molecules of ATP; red dots, molecules of adenosine. (Reproduced from [114] and modified from [105], used with permission from the American Physiological Society.)

Visceral pain is one of the most common forms of pain associated with pathological conditions like renal colic, dyspepsia, inflammatory bowel disease (IBD), angina, dysmenorrhoea and interstitial cystitis. While it is generally accepted that IBD is associated with pain (see [1,2]) there are reports that in some patients with IBD, there is hyposensitivity. P2X_3 _(homomultimer) and P2X_2/3 _(heteromultimer) receptors were cloned and shown to be largely located on small nociceptive sensory neurons in the dorsal root ganglia (DRG) in 1995 [3,4]. A schematic showing the initiation of nociception by ATP on primary afferent fibres in the periphery and purinergic relay pathways in the spinal cord are shown in Figure 1.

**Figure 2:**
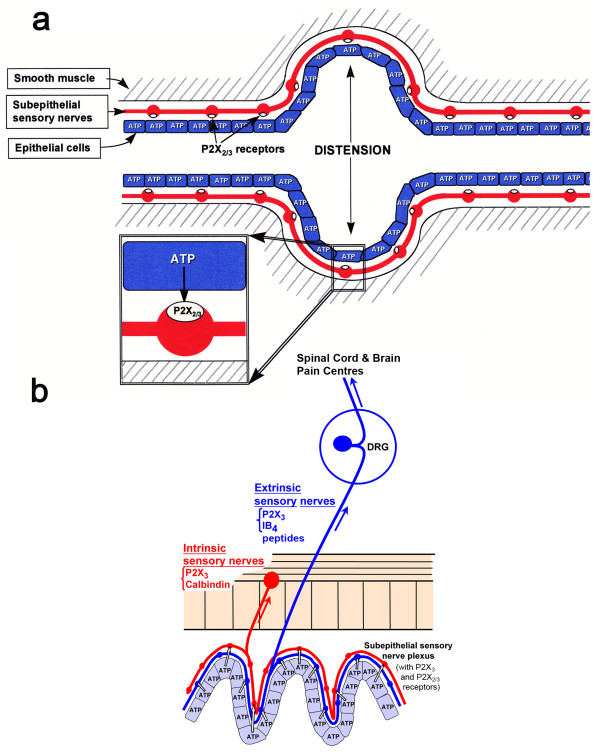
**A. Schematic representation of hypothesis for purinergic mechanosensory transduction in tubes (e.g., ureter, vagina, salivary and bile ducts, gut) and sacs (e.g., urinary and gall bladders, lung)**. It is proposed that distension leads to release of ATP from epithelium lining the tube or sac, which then acts on P2X_3 _and/or P2X_2/3 _receptors on subepithelial sensory nerves to convey sensory/nociceptive information to the CNS. (Reproduced from [5], with permission from Blackwell.) **B**. schematic of a novel hypothesis about purinergic mechanosensory transduction in the gut. It is proposed that ATP released from mucosal epithelial cells during moderate distension acts preferentially on P2X_3 _and/or P2X_2/3 _receptors on low-threshold subepithelial intrinsic sensory nerve fibers (labelled with calbindin) to modulate peristaltic reflexes. ATP released during extreme (colic) distension also acts on P2X_3 _and/or P2X_2/3 _receptors on high-threshold extrinsic sensory nerve fibers [labelled with isolectin B4 (IB4) or are peptidergic] that send messages via the dorsal root ganglia (DRG) to pain centres in the CNS. (Reproduced and modified from [115], published by John Wiley and Sons, Inc.)

A hypothesis was proposed that purinergic mechanosensory transduction occurred in visceral tubes and sacs, including ureter, bladder and gut, where ATP released from epithelial cells during distension acted on P2X_3 _homomeric and P2X_2/3 _heteromeric receptors on subepithelial sensory nerves initiating impulses in sensory pathways to pain centres in the central nervous system (CNS) [5] (Figure 2a). Evidence supporting this hypothesis in various organs is reviewed below.

### Urinary bladder

Early evidence for ATP release from rabbit urinary bladder epithelial cells by hydrostatic pressure changes was presented by Ferguson et al. [6], who speculated about this being the basis of a sensory mechanism. Prolonged exposure to a desensitizing concentration of α,β-methylene ATP (α,β-meATP) significantly reduced the activity of mechanosensitive pelvic nerve afferents in an *in vitro *model of rat urinary bladder [7]. Later, it was shown that mice lacking the P2X_3 _receptor exhibited reduced inflammatory pain and marked urinary bladder hyporeflexia with reduced voiding frequency and increased voiding volume, suggesting that P2X_3 _receptors are involved in mechanosensory transduction underlying both inflammatory pain and physiological voiding reflexes [8]. Subsequently, using P2X_2 _knockout mice and P2X_2_/P2X_3 _double knockout mice, a role for the P2X_2 _subtype was shown to be involved in mediating the sensory effect of ATP [9]. In a systematic study of purinergic mechanosensory transduction in the mouse urinary bladder, ATP was shown to be released from urothelial cells during distension, and activity initiated in pelvic sensory nerves was mimicked by ATP and α,β-meATP and attenuated by P2X_3 _antagonists as well as in P2X_3 _knockout mice; P2X_3 _receptors were localized on suburothelial sensory nerve fibres [10]. It appears that the bladder sensory DRG neurons, projecting via pelvic nerves, express predominantly P2X_2/3 _heteromultimer receptors [11].

Sensory information from the urinary bladder is conveyed by both lumbar splanchnic (LSN) and sacral pelvic (PN) nerves to the spinal cord. A study comparing the mechanosensitive properties of single afferent fibres in these two pathways showed that both low and high threshold stretch-sensitive afferents were present in both pathways [12]. Single unit analysis of sensory fibres in the mouse urinary bladder revealed both low- and high-threshold fibres sensitive to ATP contributing to physiological (non-nociceptive) and nociceptive mechanosensory transduction, respectively [13]. It was also shown that purinergic agonists increase the excitability of afferent fibres to distension. The roles of ATP released from urothelial cells and suburothelial myofibroblasts on various bladder functions have been considered at length in several reviews [14,15], and evidence presented that urothelial-released ATP alters afferent nerve excitability [16]. Amiloride, a blocker of epithelial Na^+ ^channels, has been shown to suppress ATP release from cultured urothelial cells by a hypotonic (mechanical) stimulus [17] or by stretch of intact bladder [18]. Raising the intracellular Ca^2+ ^concentration inhibits stimulation-evoked ATP release from urothelial cells [19].

ATP given intravesically stimulates the micturition reflex in awake freely moving rats, probably by stimulating suburothelial C-fibres, although other mediators are likely to be involved [20]. Studies of resiniferatoxin desensitization of capsaicin-sensitive afferents on detrusor overactivity induced by intravesicle ATP in conscious rats supported the view that increased extracellular ATP has a role in mechanosensory transduction and that ATP-induced facilitation of the micturition reflex is mediated, at least partly, by nerves other than capsaicin-sensitive afferents [8,21]. ATP has also been shown to induce a dose-dependent hypereflexia in conscious and anesthetized mice, largely via capsaicin-sensitive C-fibres; these effects were dose-dependently inhibited by pyridoxalphosphate-6-azonphenyl-2',4'-disulfonic acid (PPADS) and 2',3'-O-(2,4,6-trinitrophenyl)-ATP (TNP-ATP) [22] (Figure 2a). P2X_1 _and P2X_3 _receptors play a fundamental role in the micturition reflex in female urethane-anesthetized rats; P2X_3 _receptor blockade by phenol red raised the pressure and volume thresholds for the reflex, while P2X_1 _receptor blockade diminished motor activity associated with voiding [23]. In TRPV1 receptor knock-out mice, release of ATP is significantly depressed [24] and afferent sensitivity to distension is attenuated, especially those effects mediated by low threshold fibres related to the micturition reflex, rather than the high threshold nociceptive fibres [25].

Four functionally distinct populations of bladder sensory neurons were identified with electrophysiological recordings when guinea-pig bladder was subjected to a range of mechanical stimuli (stretch, von Frey hair stroking and focal compression of receptive fields) and chemical stimuli (α,β-methylene ATP and capsaicin) [26]. Four different major classes of extrinsic sensory C fibres have been identified in the guinea-pig bladder: one mediates muscle mechanoresponses and was unaffected by removal of the urothelium; another was activated by stretch and α,β-meATP and was reduced by urothelial removal; the third were stretch insensitive, but could be activated by mucosal stroking with von Frey hairs or α,β-meATP and reduced by urothelium removal; while the fourth class were stretch insensitive, but could be weakly activated by mucosal stroking, but not by α,β-meATP [26].

Despite the compelling evidence in support of purinergic mechanosensory transduction from several independent laboratories (including stimulation by α,β-meATP of 2 of the 4 sensory afferents classes described by Zagorodnyuk et al. [26]), a recent paper from this group claims that urothelial release of ATP and stimulation of sensory fibres is not involved in mechanosensory transduction in the bladder, but that benzamil-sensitive stretch-activated ion channels are more likely to be involved [27]. Further experiments will hopefully resolve this issue.

In rats with detrusor overactivity induced by bladder outlet obstruction, there is an increase in expression of muscarinic receptors and an increase, but to a smaller extent, of P2X_3 _receptor immunostaining [28]. Cyclophosphamide-induced bladder inflammation (a model for interstitial cystitis), sensitizes and enhances P2X_3 _and P2X_2/3 _receptor function in rat bladder sensory neurons [29]. Botulinum toxin A, which has antinociceptive effects in treating interstitial cystitis, inhibits distension-mediated urothelial release of ATP in conditions of bladder inflammation [30] as well as ATP release as a cotransmitter with acetylcholine from parasympathetic nerves [31].

In summary, there is now strong evidence from several different laboratories that ATP is released from urothelial cells during distension of the bladder wall. The ATP then activates sensory nerve endings beneath the urothelium, via P2X_3 _and P2X_2/3 _receptors, that leads, via low threshold fibres, to modulation of the voiding reflex and via high threshold fibres to reach pain centres in the CNS.

### Ureter

**Figure 3:**
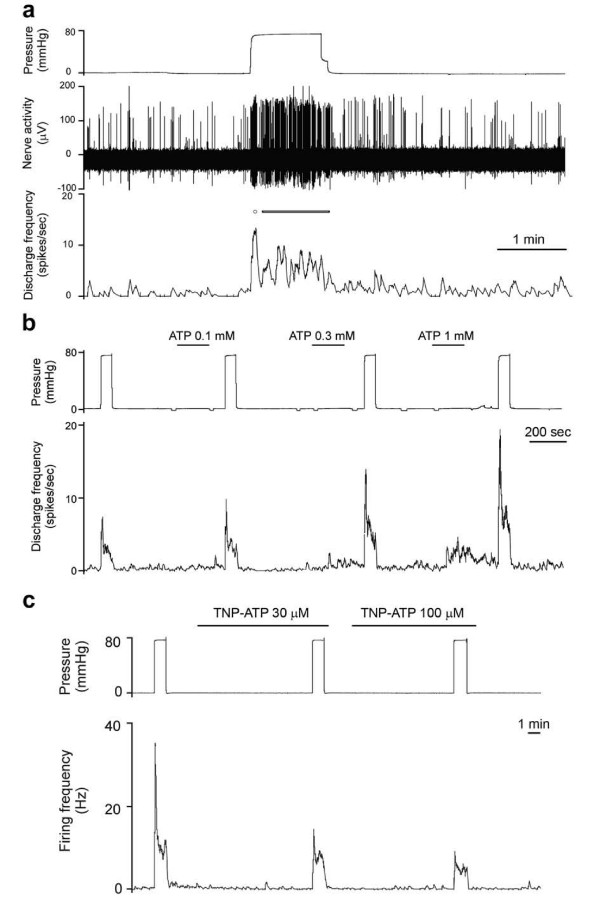
**A. Spontaneous and distension-induced activity in ureter afferent fibres**. Multifibre afferent responses to rapid distension. Note that background afferent activity occurs in bursts and that ureter distension results in an initial burst of discharge (circle) followed by a phase of maintained activity (bar). **B**. ATP can sensitise ureter afferent fibres. An example representative of distension-induced afferent activity before and following intraluminal application of increasing concentrations of ATP. **c**. TNP-ATP inhibits distension-induced afferent activity. A multifibre recording to show distension-induced afferent activity in control and in the presence of TNP-ATP. (Reproduced from [33], with permission of Elsevier.)

The uroteric colic that is induced by the passage of a kidney stone causes severe pain. Immunostaining of P2X_3 _receptors in sensory nerves in the subepithelial region was reported [32]. Multifibre recordings of ureter afferent nerves were made using a guinea pig preparation perfused *in vitro *[33]. Distension of the guinea-pig ureter increased spike discharge in sensory neurons, which was mimicked by ATP and reduced by ATP antagonists [33] (Figure 3a). The afferent responses consisted of both fast and slow components. The P2 receptor antagonists TNP-ATP and PPADS reduced distension-induced afferent activity (Figure 3b) and blocked the rapid and reduced the slower response to ATP, while the remaining responses were blocked by the selective A_1 _receptor antagonist 8-cyclopentyl-1,3-dipropylxanthine. The ecto-ATPase inhibitor (ARL-67156) produced an increase in base-line and distension-induced sensory discharge.

**Figure 4:**
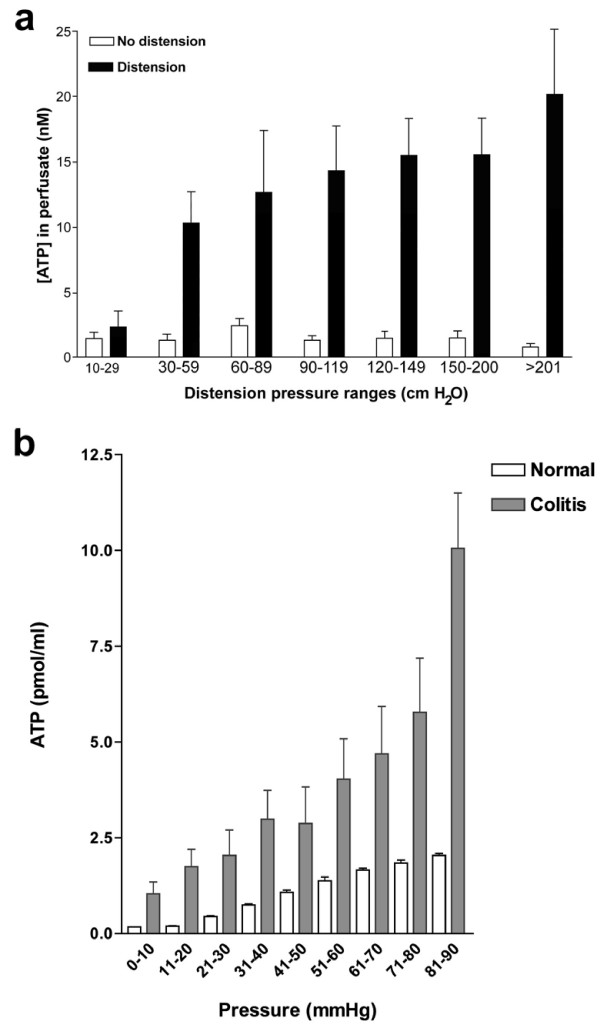
**A. ATP concentration ([ATP]) in perfusate immediately before and after distension of the human ureter, grouped in pressure ranges**. The mean [ATP] after distension is significantly greater than before distension in each pressure range P < 0.01; n = 7, error bars represent s.e.m. (Reproduced from [35], with permission from Springer.) **B**. ATP concentration in luminal fluid samples from normal and inflamed rat colorectum during distension. Values are means ± SE. (Reproduced from [67] and used with permission from the American Physiological Society.)

Knight et al. [34] found that distending the perfused guinea-pig ureter at pressures from 20-700 cm H_2_O caused a pressure-dependent release of ATP from urothelial cells, approximately 10 times the basal release levels. The ATP release was abolished by removal of the urothelium and scanning electronmicroscopy confirmed an intact urothelium after distension. ATP was not released due to activation of stretch-activated channels since gadolinium failed to affect ATP release, nor did glibenclamide, known to inhibit ATP-binding cassette proteins. However, both monensin and brefeldin A, which interfere with vesicular formation and trafficking, inhibited distension-evoked ATP release, which was Ca^2+^-dependent, indicating that ATP release from ureter urothelium might be largely mediated by vesicular exocytosis. In a recent study in our laboratory, experiments have been carried out to show that ATP is released from the human ureter upon distension (Figure 4a) and that human ureteric suburothelial sensory nerves express P2X_3 _receptors [35].

The release of ATP only occurred above a threshold of 25-30 com H_2_O. This is similar to the uroteric pressure threshold for pain measured by Risholm [36]. In a recent review of the physiology and pharmacology of the human ureter, it was suggested that purinergic receptors might be target analgesics for the treatment of ureteral colicky pain and that an additional advantage might be facilitating spontaneous ureteral stone passage [37].

### Gut

A hypothesis was proposed suggesting that purinergic mechanosensory transduction in the gut initiated both physiological reflex modulation of peristalsis via intrinsic sensory fibres and nociception via extrinsic sensory fibres [38,39] (Figure 2b). Evidence in support of this hypothesis was obtained from a rat pelvic sensory nerve-colorectal preparation [40]. Distension of the colorectum led to pressure-dependent increase in release of ATP from mucosal epithelial cells (Figure 4b) and also evoked pelvic nerve excitation. This excitation was mimicked by application of ATP and α,β-meATP and attenuated by the selective P2X_3 _and P2X_2/3 _antagonist TNP-ATP and by PPADS. The sensory discharge was potentiated by ARL-67156, an ATPase inhibitor. Single fibres analysis showed that high-threshold fibres were particularly affected by α,β-meATP. In addition to release of ATP from mucosal epithelial cells in the rat gut in response to distension (see [40]), ATP has also been shown to be released from human intestinal epithelial cells in response to osmotic swelling [41,42]. The interactions of ATP with other mediators that activate pelvic afferent fibres in the rat colorectum, including capsaicin, 5-hydroxytryptamine (5-HT), bradykinin, prostaglandins and substance P (SP), have been described [43,44]. In addition, TRPV1 channels are activated and sensitised by ATP that is released during distension [45,46], especially in pathological states such as colitis [47-49]. Carvacral, an agonist for TRPV3 channels, caused increased ATP release from colonic epithelial cells [50] and TRPV4 channels have also been shown to mediate stretch-release of ATP from urothelial cells [51]. LSN and PN nerves convey different mechanosensory information from the colon to the spinal cord. Forty percent of LSN afferents responded to α,β-meATP compared with only 7% of PN afferents [52].

Purinergic mechanosensory transduction has been described in other regions of the gastrointestinal tract. For instance, α,β-meATP was shown to stimulate mechanosensitive mucosal and tension receptors in mouse stomach and oesophagus leading to activity in vagal afferent nerves [53]. The sensitizing effects of P2X_3 _receptor agonists on mechanosensory function are induced in oesophagitis [54]. Vagal nodose (placode-derived) nociceptive fibres in guinea-pig oesophagus are exclusively C-fibres sensitive to P2X_3 _receptor agonists and rarely express SP, while jugular (neural crest-derived) nociceptive fibres include both A- and C-fibres and are insensitive to P2X_3 _agonists and mostly express SP [55]. Adenosine has been claimed to activate a subset of nociceptive vagal sensory nerves in guinea-pig oesophagus [56]. Visceral hypersensitivity may play a role in the pathogenesis of functional chest pain claimed to be of oesophageal origin. Theophylline ameliorated chest pain in 7 out of 8 patients in a clinical trial, perhaps by reducing adenosine-mediated nociception [57]. Purinergic mechanosensory transduction has also been implicated in reflex control of intestinal secretion, whereby ATP released from mucosal epithelial cells acts on P2Y_1 _receptors on enterochromaffin cells to release 5-HT (and ATP, which is stored and co-released with 5-HT from enterochromaffin cells [58]), which leads to regulation of secretion either directly or via intrinsic reflex activity [59].

Subepithelial fibroblasts in intestinal villi are highly sensitive to mechanical stimulation and release ATP during touch or stretch and probably act as mechanosensors [60]. The ATP released activates P2Y_1 _receptors on surrounding cells, which leads to intercellular propagation of Ca^2+ ^waves and contractions in networks of subepithelial fibroblasts and a signal to sensory nerve terminals in the villi [61]. Intrinsic enteric sensory nerves express P2X_3 _and P2X_2/3 _receptors [62-66]. In P2X_2 _or P2X_3 _knock-out mice, intraluminal pressure-induced peristalsis is inhibited [65,66].

ATP release and P2X_3 _and P2X_2/3 _receptor-mediated nociceptive sensory nerve responses were enhanced in a model of colitis consisting of administration to adult rats of an intrarectal enema of 30% trinitro benzene sulfonic acid in ethanol at a dose of 80 mg/kg body weight [67]. An increase in the number of DRG neurons supplying the colorectum expressing P2X_3 _receptors was also claimed and there was also a substantial increase in release of ATP with distension (Figure 4b). The excitability of visceral afferent nerves is enhanced following injury or ischemia and during inflammation, for example, in irritable bowel syndrome (IBS) [68]. Under these conditions, substances are released from various sources that often act synergistically to cause sensitization of afferent nerves to mechanical or chemical stimuli. Receptors to these substances (including ATP) represent potential targets for drug treatment aimed at attenuating the inappropriate visceral sensation and subsequent reflex activities that underlie abnormal bowel function and visceral pain (see [69,70]). Chronic functional visceral hyperalgesia induced in a rat model for IBS, induced by colonic injection of 0.5% acetic acid, is associated with potentiation of ATP-evoked responses and an enhanced expression of P2X_3 _receptors in colon-specific sensory neurons [71]. In addition, activation of spinal A_1 _receptors with adenosine, following breakdown of ATP, has been shown to modulate visceral hyperalgesia [72].

Non-erosive reflux disease shows the classic symptoms of gastro-oesophageal reflux, but in the absence of oesophageal mucosal injury. Visceral hypersensitivity plays an important role in the pathology of this disease [73]. ATP has been found to sensitise vagal afferents to mechanical stimuli in the ferret oesophagus [54] and the protein expression of P2X_3 _receptors is increased in nodose and DRG with chronic oesophageal acid exposure in a rat model [74].

### Lung

In the lung, pulmonary neuroepithelial bodies (NEBs) and more recently subepithelial receptor-like endings associated with smooth muscle (SMARs) [75] have been shown to serve as sensory organs in the lung, and P2X_3_and P2X_2/3 _receptors are expressed on a subpopulation of vagal sensory fibres that supply NEBs and SMARs with their origin in the nodose ganglia. Quinacrine staining of NEBs indicates the presence of high concentrations of ATP in their secretory vesicles, and it has been suggested that ATP is released in response to both mechanical stimulation during high-pressure ventilation and during hypoxia [76]. NEBs are oxygen sensors especially in early development, before the carotid system has matured [77]. In a study of bronchopulmonary afferent nerve activity of a mouse isolated perfused nerve-lung preparation, it was found that C fibres could be subdivided into two groups: fibres that conduct action potentials at < 0.7 ms^-1 ^and are responsive to capsaicin, bradykinin and ATP; and fibres that conduct action potentials on an average of 0.9 ms^-1 ^and respond vigorously to ATP, but not to capsaicin or bradykinin [78]. Both the TRPV1 receptor and P2X receptors mediate the sensory transduction of pulmonary reactive oxygen species, especially H_2_O_2 _and OH, by capsaicin-sensitive vagal lung afferent fibres [79].

Vagal C-fibres innervating the pulmonary system are derived from cell bodies situated in two distinct vagal sensory ganglia: the jugular (superior) ganglion neurons project fibres to the extrapulmonary airways (larynx, trachea, bronchus) and the lung parenchymal tissue, while the nodose (inferior) neurons innervate primarily structures within the lungs. Nerve terminals in the lungs from both jugular and nodose ganglia responded to capsaicin and bradykinin, but only the nodose C-fibres responded to α,β-meATP. Vagal afferent purinergic signaling may be involved in the hyperactivity associated with asthma and chronic obstructive pulmonary disease [80]. Th1 and Th2 cytokines reciprocally regulate P2X_7 _receptor function, suggesting a role for P2X_7 _receptors in pulmonary diseases, particularly lung hypersensitivity associated with chronic inflammatory responses [81].

### Uterus

It has been hypothesised that tissue stress or damage in the uterine cervix during late pregnancy and parturition leads to ATP release and sensory signalling via P2X receptors [82]. In support of this proposal, these authors have shown P2X_3 _receptor immunoreactivity in axons in the cervix, in small and medium sized neurons in L6/S1 DRG and in lamina II of the L6/S1 spinal cord segments and increases in P2X_3 _receptor expression between pregnancy day 10 and parturition (day 22/23) in the rat cervix, although not in DRG or spinal cord.

### Tooth pulp

P2X_3 _and P2X_2/3 _receptors on sensory afferents in tooth pulp appear to mediate nociception [83-86], perhaps from ATP released by mechanical distension or inflammation of odontoblasts. Mustard oil application to the tooth pulp in anaesthetised rats produced long-lasting central sensitisation, reflected by increases in neuronal mechanoreceptive field size; TNP-ATP reversibly attenuated the mustard oil sensitisation for more than 15 minutes [87].

### Tongue

P2X_3 _receptors are abundantly present on sensory nerve terminals in the tongue [88] and ATP and α,β-meATP have been shown to excite trigeminal lingual nerve terminals in an *in vitro *preparation of intra-arterially perfused rat mimicking nociceptive responses to noxious mechanical stimulation and high temperature [89]. A purinergic mechanosensory transduction mechanism for the initiation of pain was considered. Taste sensations appear to be mediated both by P2Y_1 _receptor-activated impulses in sensory fibres in the chorda tympani [90] and by P2X_2 _and P2X_3 _and, perhaps, P2X_2/3 _receptors [91].

### Potential Therapeutic Strategies

The search is on for selective P2X_3 _and P2X_2/3 _receptor antagonists that are orally bioavailable and do not degrade *in vivo *for the treatment of pain (see [92-96]). Table 1 summarises the drugs widely available. Suramin, PPADS and Reactive blue 2 have been used as non-selective antagonists at P2X_3 _and P2X_2/3 _receptors on nociceptive sensory nerve endings. PPADS has the advantage that it associates and dissociates approximately 100 to 10,000 times more slowly than other known antagonists [97]. The trinitrophenyl-substituted nucleotide, TNP-ATP, is a very potent antagonist at both P2X_3 _and P2X_2/3 _receptors. A-317491 (synthesised by Abbott Laboratories) and compound RO3 (synthesised by Roche Palo Alto) are both effective P2X_3 _and P2X_2/3 _antagonists, the latter being orally bioavailable and stable *in vivo*. Antagonism of P2X_1 _and P2X_3 _receptors by phenol red has been reported and tetramethylpyrazine, a traditional Chinese medicine, used as an analgesic for dysmenorrhoea, was claimed to block P2X_3 _receptor signalling [98].

**Table 1:** P2X_3 _and P2X_2/3 _receptor antagonists

Antagonist	P2X_3_	P2X2/3
Suramin and analogues NF449, NF110	√	√
PPADS and derivatives MRS2159 & MRS2257	√√	√
Reactive blue 2 and derivatives TNP-ATP	√	√
A-317491 (selective)	√√√	√√√
Phenol red	√√√	√√√
Tetramethylpyrazine	√√	-
RO4 (orally bioavailable, stable *in vivo*)	√√	?
Ip_5_I	√√	√√
βγcarboxymethylene ATP	√√	-
βγchlorophosphnomethylene ATP	?	√√
	?	√√

From [93,95,112,113]

Antisense oligonucleotides have been used to down-regulate the P2X_3 _receptor, and in models of neuropathic (partial sciatic nerve ligation) and inflammatory (complete Freund's adjuvant pain, inhibition of the development of mechanical hyperalgesia as well as significant reversal of established hyperalgesia, were observed within 2 days of treatment [99-101]. P2X_3 _antisense oligonucleotides or antagonists appear to be less effective for treating discogenic (lumbar intervertebral disc) than cutaneous tissue pain [102]. Combined antisense and RNA interference-mediated treatment for specific inhibition of the recombinant rat P2X_3 _receptor appears to be promising for pain therapy [103]. P2X_3 _double-stranded short interfering RNA relieves chronic neuropathic pain and opens up new avenues for therapeutic pain strategies in man [104].

While P2X_3 _and P2X_2/3 _receptors, expressed in sensory neurons, were the predominant P2 receptor subtypes first recognised to be involved in the initiation of nociception (see [105,106]), it has become apparent more recently that P2Y receptors are also present [107] and that these are involved in modulation of pain transmission [108]. P2Y receptors appear to potentiate pain induced by chemical or physical stimuli via capsaicin sensitive TRPV1 channels and it has been proposed that the functional interaction between P2Y_2 _receptors and TRPV1 channels in nociceptors could underlie ATP-induced inflammatory pain [45]. P2Y_1 _receptor-mediated responses also enhance the sensitivity of TRPV1-mediated responses to capsaicin, protons and temperature in a protein kinase C-dependent manner [109]. ATP-induced hyperalgesia was abolished in mice lacking TRPV1 receptors.

It has been claimed that opioids inhibit purinergic nociception in rat sensory neurons and fibres via a G protein-dependent mechanism [110]. Cannabinoids act as inhibitory modulators of nociceptive responses produced by P2X_2/3 _receptors [111].

There are no publications to date describing clinical evaluations of P2 receptor antagonists and related purinergic compounds for the relief of pain, although clinical trials for some compounds are in progress (see [93,94]). Other therapeutic approaches to pain are being considered, including the development of agents that control the expression of receptors and those that enhance ATP breakdown. Further, while it is now clear that many different cell types release ATP physiologically in response to mechanical distortion, hypoxia, and various agents, we still await clear understanding of the mechanisms that underlie ATP transport. Hopefully, when this becomes clearer, agents will be developed that will be able to inhibit ATP release, another useful way forward as a therapeutic strategy.

## Conclusion

Compelling evidence has been presented for the role of purinergic mechanosensory transduction where ATP, released from epithelial cells lining the bladder, ureter and gut during distension, acts on P2X_3 _and/or P2X_2/3 _receptors on subepithelial sensory nerve terminals to relay nociceptive messages via sensory ganglia and spinal cord to pain centres in the CNS.

Antagonists to P2X_3 _and P2X_2/3 _receptors are being explored to treat visceral pain and the possibilities for development of agents that inhibit ATP transport from epithelial cells or enhance ATP breakdown after its release are discussed.

## Competing interests

The author declares that he has no competing interests.

## References

[B1] Bueno L Gastrointestinal pharmacology: irritable bowel syndrome Curr Opin Pharmacol 2005 5 583 588 10.1016/j.coph.2005.06.006 16188501

[B2] Kraneveld AD Rijnierse A Nijkamp FP Garssen J Neuro-immune interactions in inflammatory bowel disease and irritable bowel syndrome: future therapeutic targets Eur J Pharmacol 2008 585 361 374 10.1016/j.ejphar.2008.02.095 18417115

[B3] Chen CC Akopian AN Sivilotti L Colquhoun D Burnstock G Wood JN A P2X purinoceptor expressed by a subset of sensory neurons Nature 1995 377 428 431 10.1038/377428a0 7566119

[B4] Lewis C Neidhart S Holy C North RA Buell G Surprenant A Coexpression of P2X_2 _and P2X_3 _receptor subunits can account for ATP-gated currents in sensory neurons Nature 1995 377 432 435 10.1038/377432a0 7566120

[B5] Burnstock G Release of vasoactive substances from endothelial cells by shear stress and purinergic mechanosensory transduction J Anat 1999 194 335 342 10.1046/j.1469-7580.1999.19430335.x 10386771 PMC1467933

[B6] Ferguson DR Kennedy I Burton TJ ATP is released from rabbit urinary bladder epithelial cells by hydrostatic pressure changes - a possible sensory mechanism? J Physiol 1997 505 503 511 10.1111/j.1469-7793.1997.503bb.x 9423189 PMC1160080

[B7] Namasivayam S Eardley I Morrison JFB Purinergic sensory neurotransmission in the urinary bladder: an *in vitro *study in the rat BJU Int 1999 84 854 860 10.1046/j.1464-410x.1999.00310.x 10532986

[B8] Cockayne DA Hamilton SG Zhu Q-M Dunn PM Zhong Y Novakovic S Malmberg AB Cain G Berson A Kassotakis L Hedley L Lachnit WG Burnstock G McMahon SB Ford APDW Urinary bladder hyporeflexia and reduced pain-related behaviour in P2X_3_-deficient mice Nature 2000 407 1011 1015 10.1038/35039519 11069181

[B9] Cockayne DA Dunn PM Zhong Y Hamilton SG Cain GR Knight G Ruan H-Z Ping Y Nunn P Bei M McMahon SB Burnstock G Ford APDW P2X_2 _knockout mice and P2X_2_/P2X_3 _double knockout mice reveal a role for the P2X_2 _receptor subunit in mediating multiple sensory effects of ATP J Physiol 2005 567 621 639 10.1113/jphysiol.2005.088435 15961431 PMC1474198

[B10] Vlaskovska M Kasakov L Rong W Bodin P Bardini M Cockayne DA Ford APDW Burnstock G P2X_3 _knockout mice reveal a major sensory role for urothelially released ATP J Neurosci 2001 21 5670 5677 11466438 10.1523/JNEUROSCI.21-15-05670.2001 PMC6762653

[B11] Zhong Y Banning AS Cockayne DA Ford APDW Burnstock G McMahon SB Bladder and cutaneous sensory neurons of the rat express different functional P2X receptors Neuroscience 2003 120 667 675 10.1016/S0306-4522(03)00243-4 12895508

[B12] Xu L Gebhart GF Characterization of mouse lumbar splanchnic and pelvic nerve urinary bladder mechanosensory afferents J Neurophysiol 2008 99 244 253 10.1152/jn.01049.2007 18003875 PMC2659401

[B13] Rong W Spyer M Burnstock G Activation and sensitisation of low and high threshold afferent fibres mediated by P2X receptors in the mouse urinary bladder J Physiol 2002 541 591 600 10.1113/jphysiol.2001.013469 12042363 PMC2290323

[B14] Lazzeri M The physiological function of the urothelium - more than a simple barrier Urol Int 2006 76 289 295 10.1159/000092049 16679827

[B15] Sui GP Wu C Fry CH Characterization of the purinergic receptor subtype on guinea-pig suburothelial myofibroblasts BJU Int 2006 97 1327 1331 10.1111/j.1464-410X.2006.06200.x 16686733

[B16] de Groat WC Integrative control of the lower urinary tract: preclinical perspective Br J Pharmacol 2006 147 Suppl 2 S25 S40 10.1038/sj.bjp.0706604 16465182 PMC1751498

[B17] Birder LA Barrick SR Roppolo JR Kanai AJ de Groat WC Kiss S Buffington CA Feline interstitial cystitis results in mechanical hypersensitivity and altered ATP release from bladder urothelium Am J Physiol Renal Physiol 2003 285 F423 F429 12759226 10.1152/ajprenal.00056.2003

[B18] Du S Araki I Mikami Y Zakoji H Beppu M Yoshiyama M Takeda M Amiloride-sensitive ion channels in urinary bladder epithelium involved in mechanosensory transduction by modulating stretch-evoked adenosine triphosphate release Urology 2007 69 590 595 10.1016/j.urology.2007.01.039 17382185

[B19] Matsumoto-Miyai K Kagase A Murakawa Y Momota Y Kawatani M Extracellular Ca^2+ ^regulates the stimulus-elicited ATP release from urothelium Auton Neurosci 2009 150 94 99 10.1016/j.autneu.2009.05.253 19525154

[B20] Pandita RK Andersson KE Intravesical adenosine triphosphate stimulates the micturition reflex in awake, freely moving rats J Urol 2002 168 1230 1234 10.1016/S0022-5347(05)64631-9 12187273

[B21] Brady CM Apostolidis A Yiangou Y Baecker PA Ford AP Freeman A Jacques TS Fowler CJ Anand P P2X_3_-immunoreactive nerve fibres in neurogenic detrusor overactivity and the effect of intravesical resiniferatoxin Eur Urol 2004 46 247 253 10.1016/j.eururo.2003.12.017 15245821

[B22] Hu ST Gever J Nunn PA Ford AP Zhu Q-M Cystometric studies with ATP, PPADS and TNP-ATP in conscious and anaesthetised C57BL/6 mice J Urol 2004 171 461 462 10.1097/01.ju.0000107964.61300.f6

[B23] King BF Knowles I Burnstock G Ramage A Investigation of the effects of P2 purinoceptor ligands on the micturition reflex in female urethane-anaesthetised rats Br J Pharmacol 2004 142 519 530 10.1038/sj.bjp.0705790 15148261 PMC1574961

[B24] Birder LA Nakamura Y Kiss S Nealen ML Barrick S Kanai AJ Wang E Ruiz G de Groat WC Apodaca G Watkins S Caterina MJ Altered urinary bladder function in mice lacking the vanilloid receptor TRPV1 Nat Neurosci 2002 5 856 860 10.1038/nn902 12161756

[B25] Daly D Rong W Chess-Williams R Chapple C Grundy D Bladder afferent sensitivity in wild-type and TRPV1 knockout mice J Physiol 2007 583 663 674 10.1113/jphysiol.2007.139147 17627983 PMC2277033

[B26] Zagorodnyuk VP Gibbins IL Costa M Brookes SJ Gregory SJ Properties of the major classes of mechanoreceptors in the guinea pig bladder J Physiol 2007 585 147 163 10.1113/jphysiol.2007.140244 17916614 PMC2375472

[B27] Zagorodnyuk VP Brookes SJ Spencer NJ Gregory S Mechanotransduction and chemosensitivity of two major classes of bladder afferents with endings in the vicinity to the urothelium J Physiol 2009 587 3523 3538 10.1113/jphysiol.2009.172577 19470774 PMC2742279

[B28] Kim JC Yoo JS Park EY Hong SH Seo SI Hwang TK Muscarinic and purinergic receptor expression in the urothelium of rats with detrusor overactivity induced by bladder outlet obstruction BJU Int 2008 101 371 375 10.1111/j.1464-410X.2007.07251.x 17922866

[B29] Dang K Lamb K Cohen M Bielefeldt K Gebhart GF Cyclophosphamide-induced bladder inflammation sensitizes and enhances P2X receptor function in rat bladder sensory neurons J Neurophysiol 2008 99 49 59 10.1152/jn.00211.2007 17959738 PMC2659400

[B30] Smith CP Vemulakonda VM Kiss S Boone TB Somogyi GT Enhanced ATP release from rat bladder urothelium during chronic bladder inflammation: effect of botulinum toxin A Neurochem Int 2005 47 291 297 10.1016/j.neuint.2005.04.021 15970360

[B31] Mackenzie I Burnstock G Dolly JO The effects of purified botulinum neurotoxin type A on cholinergic, adrenergic and non-adrenergic, atropine-resistant autonomic neuromuscular transmission Neuroscience 1982 7 997 1006 10.1016/0306-4522(82)90056-2 6124898

[B32] Lee HY Bardini M Burnstock G Distribution of P2X receptors in the urinary bladder and the ureter of the rat J Urol 2000 163 2002 2007 10.1016/S0022-5347(05)67618-5 10799247

[B33] Rong W Burnstock G Activation of ureter nociceptors by exogenous and endogenous ATP in guinea pig Neuropharmacology 2004 47 1093 1101 10.1016/j.neuropharm.2004.08.003 15555643

[B34] Knight GE Bodin P de Groat WC Burnstock G ATP is released from guinea pig ureter epithelium on distension Am J Physiol Renal Physiol 2002 282 F281 F288 11788442 10.1152/ajprenal.00293.2000

[B35] Calvert RC Thompson CS Burnstock G ATP release from the human ureter on distension and P2X_3 _receptor expression on suburothelial sensory nerves Purinergic Signalling 2008 4 377 381 10.1007/s11302-008-9123-1 18819020 PMC2583211

[B36] Risholm L Studies on renal colic and its treatment by posterior splanchnic block Acta Chir Scand 1954 184 Suppl 5 64 13137835

[B37] Canda AE Turna B Cinar GM Nazli O Physiology and pharmacology of the human ureter: basis for current and future treatments Urol Int 2007 78 289 298 10.1159/000100830 17495484

[B38] Burnstock G Abbracchio MP, Williams M Purinergic signalling in gut Handbook of Experimental Pharmacology. Purinergic and Pyrimidinergic Signalling II - Cardiovascular, Respiratory, Immune, Metabolic and Gastrointestinal Tract Function 2001 151/II Berlin: Springer-Verlag 141 238

[B39] Burnstock G Purine-mediated signalling in pain and visceral perception Trends Pharmacol Sci 2001 22 182 188 10.1016/S0165-6147(00)01643-6 11282418

[B40] Wynn G Rong W Xiang Z Burnstock G Purinergic mechanisms contribute to mechanosensory transduction in the rat colorectum Gastroenterology 2003 125 1398 1409 10.1016/j.gastro.2003.07.008 14598256

[B41] Dezaki K Tsumura T Maeno E Okada Y Receptor-mediated facilitation of cell volume regulation by swelling-induced ATP release in human epithelial cells Jpn J Physiol 2000 50 235 241 10.2170/jjphysiol.50.235 10880880

[B42] Wijk T van der Tomassen SF Houtsmuller AB De Jonge HR Tilly BC Increased vesicle recycling in response to osmotic cell swelling. Cause and consequence of hypotonicity-provoked ATP release J Biol Chem 2003 278 40020 40025 10.1074/jbc.M307603200 12871943

[B43] Barthó L Lénárd LJ Lázár Z Maggi CA Connections between P2 purinoceptors and capsaicin-sensitive afferents in the intestine and other tissues Eur J Pharmacol 1999 375 203 210 10.1016/S0014-2999(99)00253-8 10443576

[B44] Wynn G Burnstock G Adenosine 5'-triphosphate and it's relationship with other mediators that activate pelvic afferent neurons in the rat colorectum Purinergic Signalling 2006 2 517 526 10.1007/s11302-005-5305-2 18404489 PMC2104004

[B45] Lakshmi S Joshi PG Co-activation of P2Y_2 _receptor and TRPV channel by ATP: implications for ATP induced pain Cell Mol Neurobiol 2005 25 819 832 10.1007/s10571-005-4936-8 16133936 PMC11529488

[B46] Christianson JA Bielefeldt K Altier C Cenac N Davis BM Gebhart GF High KW Kollarik M Randich A Undem B Vergnolle N Development, plasticity and modulation of visceral afferents Brain Res Rev 2009 60 171 186 10.1016/j.brainresrev.2008.12.004 19150371 PMC2841801

[B47] Sugiura T Bielefeldt K Gebhart GF Mouse colon sensory neurons detect extracellular acidosis via TRPV1 Am J Physiol Cell Physiol 2007 292 C1768 C1774 10.1152/ajpcell.00440.2006 17251322

[B48] De Schepper HU De Winter BY Van Nassauw L Timmermans JP Herman AG Pelckmans PA De Man JG TRPV1 receptors on unmyelinated C-fibres mediate colitis-induced sensitization of pelvic afferent nerve fibres in rats J Physiol 2008 586 5247 5258 10.1113/jphysiol.2008.159731 18755744 PMC2652143

[B49] Malin SA Christianson JA Bielefeldt K Davis BM TPRV1 expression defines functionally distinct pelvic colon afferents J Neurosci 2009 29 743 752 10.1523/JNEUROSCI.3791-08.2009 19158300 PMC2790201

[B50] Ueda T Yamada T Ugawa S Ishida Y Shimada S TRPV3, a thermosensitive channel is expressed in mouse distal colon epithelium Biochem Biophys Res Commun 2009 383 130 134 10.1016/j.bbrc.2009.03.143 19336223

[B51] Mochizuki T Sokabe T Araki I Fujishita K Shibasaki K Uchida K Naruse K Koizumi S Takeda M Tominaga M The TRPV4 cation channel mediates stretch-evoked Ca^2+ ^influx and ATP release in primary urothelial cell cultures J Biol Chem 2009 284 21257 21264 10.1074/jbc.M109.020206 19531473 PMC2755849

[B52] Brierley SM Carter R Jones W III Xu L Robinson DR Hicks GA Gebhart GF Blackshaw LA Differential chemosensory function and receptor expression of splanchnic and pelvic colonic afferents in mice J Physiol 2005 567 267 281 10.1113/jphysiol.2005.089714 15946967 PMC1474170

[B53] Page AJ Martin CM Blackshaw LA Vagal mechanoreceptors and chemoreceptors in mouse stomach and esophagus J Neurophysiol 2002 87 2095 2103 11929927 10.1152/jn.00785.2001

[B54] Page AJ O'Donnell TA Blackshaw LA P2X purinoceptor-induced sensitization of ferret vagal mechanoreceptors in oesophageal inflammation J Physiol 2000 523 403 411 10.1111/j.1469-7793.2000.00403.x 10699084 PMC2269809

[B55] Yu S Undem BJ Kollarik M Vagal afferent nerves with nociceptive properties in guinea-pig oesophagus J Physiol 2005 563 831 842 10.1113/jphysiol.2004.079574 15649987 PMC1665603

[B56] Ru F Kollarik M Adenosine activates a subset of nociceptive vagal sensory nerves in oesophagus [abstract] Gastroenterology 2006 130 A252

[B57] Rao SS Mudipalli RS Mujica V Utech CL Zhao X Conklin JL An open-label trial of theophylline for functional chest pain Dig Dis Sci 2002 47 2763 2768 10.1023/A:1021017524660 12498299

[B58] Winkler H Westhead E The molecular organization of adrenal chromaffin granules Neuroscience 1980 5 1803 1823 10.1016/0306-4522(80)90031-7 7432623

[B59] Cooke HJ Wunderlich J Christofi FL "The force be with you": ATP in gut mechanosensory transduction News Physiol Sci 2003 18 43 49 12644618 10.1152/nips.01411.2002

[B60] Furuya K Sokabe M Furuya S Characteristics of subepithelial fibroblasts as a mechano-sensor in the intestine: cell-shape-dependent ATP release and P2Y1 signaling J Cell Sci 2005 118 3289 3304 10.1242/jcs.02453 16030139

[B61] Furuya S Furuya K Subepithelial fibroblasts in intestinal villi: roles in intercellular communication Int Rev Cytol 2007 264 165 223 full_text 17964923 10.1016/S0074-7696(07)64004-2

[B62] Bertrand PP Bornstein JC ATP as a putative sensory mediator: activation of intrinsic sensory neurons of the myenteric plexus via P2X receptors J Neurosci 2002 22 4767 4775 12077173 10.1523/JNEUROSCI.22-12-04767.2002 PMC6757757

[B63] Castelucci P Robbins HL Poole DP Furness JB The distribution of purine P2X_2 _receptors in the guinea-pig enteric nervous system Histochem Cell Biol 2002 117 415 422 10.1007/s00418-002-0404-4 12029488

[B64] Poole DP Castelucci P Robbins HL Chiocchetti R Furness JB The distribution of P2X_3 _purine receptor subunits in the guinea pig enteric nervous system Auton Neurosci 2002 101 39 47 10.1016/S1566-0702(02)00179-0 12462358

[B65] Bian X Ren J DeVries M Schnegelsberg B Cockayne DA Ford AP Galligan JJ Peristalsis is impaired in the small intestine of mice lacking the P2X_3 _subunit J Physiol 2003 551 309 322 10.1113/jphysiol.2003.044172 12813150 PMC2343160

[B66] Ren J Bian X DeVries M Schnegelsberg B Cockayne DA Ford AP Galligan JJ P2X_2 _subunits contribute to fast synaptic excitation in myenteric neurons of the mouse small intestine J Physiol 2003 552 809 821 10.1113/jphysiol.2003.047944 12937291 PMC2343442

[B67] Wynn G Bei M Ruan H-Z Burnstock G Purinergic component of mechanosensory transduction is increased in a rat model of colitis Am J Physiol Gastrointest Liver Physiol 2004 287 G647 G657 10.1152/ajpgi.00020.2004 15331354

[B68] Shinoda M Feng B Gebhart GF Peripheral and central P2X_3 _receptor contributions to colon mechanosensitivity and hypersensitivity in the mouse Gastroenterology in press 19549524 10.1053/j.gastro.2009.06.048 PMC2789894

[B69] Kirkup AJ Brunsden AM Grundy D Receptors and transmission in the brain-gut axis: Potential for novel therapies. I. Receptors on visceral afferents Am J Physiol Gastrointest Liver Physiol 2001 280 G787 G794 11292585 10.1152/ajpgi.2001.280.5.G787

[B70] Holzer P Gastrointestinal pain in functional bowel disorders: sensory neurons as novel drug targets Expert Opin Ther Targets 2004 8 107 123 10.1517/14728222.8.2.107 15102553

[B71] Xu GY Shenoy M Winston JH Mittal S Pasricha PJ P2X receptor-mediated visceral hyperalgesia in a rat model of chronic visceral hypersensitivity Gut 2008 57 1230 1237 10.1136/gut.2007.134221 18270243

[B72] Zahn PK Straub H Wenk M Pogatzki-Zahn EM Adenosine A1 but not A2a receptor agonist reduces hyperalgesia caused by a surgical incision in rats: a pertussis toxin-sensitive G protein-dependent process Anesthesiology 2007 107 797 806 10.1097/01.anes.0000286982.36342.3f 18073555

[B73] Knowles CH Aziz Q Visceral hypersensitivity in non-erosive reflux disease Gut 2008 57 674 683 10.1136/gut.2007.127886 18079285

[B74] Banerjee B Medda BK Shaker R Sengupta JN TRPV1 and P2X3 expression in vagal and spinal pathways following acid-induced esophagitis in rats [abstract] Gastroenterology 2006 130 A133

[B75] Brouns I De Proost I Pintelon I Timmermans JP Adriaensen D Sensory receptors in the airways: neurochemical coding of smooth muscle-associated airway receptors and pulmonary neuroepithelial body innervation Auton Neurosci 2006 126-127 307 319 10.1016/j.autneu.2006.02.006 16600695

[B76] Rich PB Douillet CD Mahler SA Husain SA Boucher RC Adenosine triphosphate is released during injurious mechanical ventilation and contributes to lung edema J Trauma 2003 55 290 297 10.1097/01.TA.0000078882.11919.AF 12913640

[B77] Brouns I Van Genechten J Burnstock G Timmermans J-P Adriaensen D Ontogenesis of P2X_3 _receptor-expressing nerve fibres in the rat lung, with special reference to neuroepithelial bodies Biomedical Research 2003 14 80 86

[B78] Kollarik M Dinh QT Fischer A Undem BJ Capsaicin-sensitive and -insensitive vagal bronchopulmonary C-fibres in the mouse J Physiol 2003 551 869 879 10.1113/jphysiol.2003.042028 12909686 PMC2343302

[B79] Ruan T Lin YS Lin KS Kou YR Sensory transduction of pulmonary reactive oxygen species by capsaicin-sensitive vagal lung afferent fibres in rats J Physiol 2005 565 563 578 10.1113/jphysiol.2005.086181 15802291 PMC1464522

[B80] Adriaensen D Timmermans JP Purinergic signalling in the lung: important in asthma and COPD? Curr Opin Pharmacol 2004 4 207 214 10.1016/j.coph.2004.01.010 15140410

[B81] Lemaire I Leduc N Purinergic P2X_7 _receptor function in lung alveolar macrophages: pharmacologic characterisation and bidirectional regulation by Th1 and Th2 cytokines Drug Dev Res 2004 59 118 127 10.1002/ddr.10209

[B82] Papka RE Hafemeister J Storey-Workley M P2X receptors in the rat uterine cervix, lumbosacral dorsal root ganglia, and spinal cord during pregnancy Cell Tissue Res 2005 321 35 44 10.1007/s00441-005-1114-8 15902498

[B83] Cook SP Vulchanova L Hargreaves KM Elde R McCleskey EW Distinct ATP receptors on pain-sensing and stretch-sensing neurons Nature 1997 387 505 508 10.1038/387505a0 9168113

[B84] Alavi AM Dubyak GR Burnstock G Immunohistochemical evidence for ATP receptors in human dental pulp J Dental Res 2001 80 476 483 10.1177/00220345010800021501 11332536

[B85] Jiang J Gu J Expression of adenosine triphosphate P2X_3 _receptors in rat molar pulp and trigeminal ganglia Oral Surg Oral Med Oral Pathol Oral Radiol Endod 2002 94 622 626 10.1067/moe.2002.128973 12424458

[B86] Renton T Yiangou Y Baecker PA Ford AP Anand P Capsaicin receptor VR1 and ATP purinoceptor P2X3 in painful and nonpainful human tooth pulp J Orofac Pain 2003 17 245 250 14520770

[B87] Hu B Chiang CY Hu JW Dostrovsky JO Sessle BJ P2X receptors in trigeminal subnucleus caudalis modulate central sensitization in trigeminal subnucleus oralis J Neurophysiol 2002 88 1614 1624 12364492 10.1152/jn.2002.88.4.1614

[B88] Bo X Alavi A Xiang Z Oglesby I Ford A Burnstock G Localization of ATP-gated P2X_2 _and P2X_3 _receptor immunoreactive nerves in rat taste buds Neuroreport 1999 10 1107 1111 10.1097/00001756-199904060-00037 10321492

[B89] Rong W Burnstock G Spyer KM P2X purinoceptor-mediated excitation of trigeminal lingual nerve terminals in an *in vitro *intra-arterially perfused rat tongue preparation J Physiol 2000 524 891 902 10.1111/j.1469-7793.2000.00891.x 10790166 PMC2269894

[B90] Kataoka S Toyono T Seta Y Ogura T Toyoshima K Expression of P2Y_1 _receptors in rat taste buds Histochem Cell Biol 2004 121 419 426 10.1007/s00418-004-0647-3 15103469

[B91] Finger TE Danilova V Barrows J Bartel DL Vigers AJ Stone L Hellekant G Kinnamon SC ATP signaling is crucial for communication from taste buds to gustatory nerves Science 2005 310 1495 1499 10.1126/science.1118435 16322458

[B92] Burnstock G Purinergic P2 receptors as targets for novel analgesics Pharmacol Therap 2006 110 433 454 10.1016/j.pharmthera.2005.08.013 16226312

[B93] Gever J Cockayne DA Dillon MP Burnstock G Ford APDW Pharmacology of P2X channels Pflugers Arch 2006 452 513 537 10.1007/s00424-006-0070-9 16649055

[B94] Gever JR Rothschild S Henningsen R Martin R Hackos D Panicker S Milla ME Oglesby I Dillon MP Burnstock G Ford APDW RO-4, a novel, potent orally bioavailable P2X_3_/P2X_2/3 _antagonist Br J Pharmacol 2009 in press 10.1111/j.1476-5381.2010.00796.x PMC2938810 20590629

[B95] Carter DS Alam M Cai H Dillon MP Ford AP Gever JR Jahangir A Lin C Moore AG Wagner PJ Zhai Y Identification and SAR of novel diaminopyrimidines. Part 1: The discovery of RO-4, a dual P2X_3_/P2X_2/3 _antagonist for the treatment of pain Bioorg Med Chem Lett 2009 19 1628 1631 10.1016/j.bmcl.2009.02.003 19231180

[B96] Jahangir A Alam M Carter DS Dillon MP Bois DJ Ford AP Gever JR Lin C Wagner PJ Zhai Y Zira J Identification and SAR of novel diaminopyrimidines. Part 2: The discovery of RO-51, a potent and selective, dual P2X_3_/P2X_2/3 _antagonist for the treatment of pain Bioorg Med Chem Lett 2009 19 1632 1635 10.1016/j.bmcl.2009.01.097 19231178

[B97] Spelta V Jiang LH Surprenant A North RA Kinetics of antagonist actions at rat P2X_2/3 _heteromeric receptors Br J Pharmacol 2002 135 1524 1530 10.1038/sj.bjp.0704591 11906966 PMC1573256

[B98] Liang SD Gao Y Xu CS Xu BH Mu SN Effect of tetramethylpyrazine on acute nociception mediated by signaling of P2X receptor activation in rat Brain Res 2004 995 247 252 10.1016/j.brainres.2003.09.070 14672814

[B99] Barclay J Patel S Dorn G Wotherspoon G Moffatt S Eunson L Abdel'al S Natt F Hall J Winter J Bevan S Wishart W Fox A Ganju P Functional downregulation of P2X_3 _receptor subunit in rat sensory neurons reveals a significant role in chronic neuropathic and inflammatory pain J Neurosci 2002 22 8139 8147 12223568 10.1523/JNEUROSCI.22-18-08139.2002 PMC6758070

[B100] Honore P Mikusa J Bianchi B McDonald H Cartmell J Faltynek C Jarvis MF TNP-ATP, a potent P2X3 receptor antagonist, blocks acetic acid-induced abdominal constriction in mice: comparison with reference analgesics Pain 2002 96 99 105 10.1016/S0304-3959(01)00434-1 11932066

[B101] Stone LS Vulchanova L The pain of antisense: in vivo application of antisense oligonucleotides for functional genomics in pain and analgesia Adv Drug Deliv Rev 2003 55 1081 1112 10.1016/S0169-409X(03)00105-4 12935946

[B102] Aoki Y Ohtori S Takahashi K Ino H Ozawa T Douya H Chiba T Moriya H P2X_3_-immunoreactive primary sensory neurons innervating lumbar intervertebral disc in rats Brain Res 2003 989 214 220 10.1016/S0006-8993(03)03365-1 14556943

[B103] Hemmings-Mieszczak M Dorn G Natt FJ Hall J Wishart WL Independent combinatorial effect of antisense oligonucleotides and RNAi-mediated specific inhibition of the recombinant rat P2X3 receptor Nucleic Acids Res 2003 31 2117 2126 10.1093/nar/gkg322 12682362 PMC153750

[B104] Dorn G Patel S Wotherspoon G Hemmings-Mieszczak M Barclay J Natt FJ Martin P Bevan S Fox A Ganju P Wishart W Hall J siRNA relieves chronic neuropathic pain Nucleic Acids Res 2004 32 e49 10.1093/nar/gnh044 15026538 PMC390346

[B105] Burnstock G Wood JN Purinergic receptors: their role in nociception and primary afferent neurotransmission Curr Opin Neurobiol 1996 6 526 532 10.1016/S0959-4388(96)80060-2 8794102

[B106] Burnstock G P2X receptors in sensory neurones Br J Anaesth 2000 84 476 488 10823099 10.1093/oxfordjournals.bja.a013473

[B107] Ruan H-Z Burnstock G Localisation of P2Y_1 _and P2Y_4 _receptors in dorsal root, nodose and trigeminal ganglia of the rat Histochemistry and Cell Biology 2003 120 415 426 10.1007/s00418-003-0579-3 14564529

[B108] Gerevich Z Borvendeg SJ Schroder W Franke H Wirkner K Norenberg W Furst S Gillen C Illes P Inhibition of N-type voltage-activated calcium channels in rat dorsal root ganglion neurons by P2Y receptors is a possible mechanism of ADP-induced analgesia J Neurosci 2004 24 797 807 10.1523/JNEUROSCI.4019-03.2004 14749424 PMC6729814

[B109] Tominaga M Wada M Masu M Potentiation of capsaicin receptor activity by metabotropic ATP receptors as a possible mechanism for ATP-evoked pain and hyperalgesia Proc Natl Acad Sci USA 2001 98 6951 6956 10.1073/pnas.111025298 11371611 PMC34459

[B110] Chizhmakov I Yudin Y Mamenko N Prudnikov I Tamarova Z Krishtal O Opioids inhibit purinergic nociceptors in the sensory neurons and fibres of rat via a G protein-dependent mechanism Neuropharmacology 2005 48 639 647 10.1016/j.neuropharm.2004.12.009 15814099

[B111] Krishtal O Lozovaya N Fedorenko A Savelyev I Chizhmakov I The agonists for nociceptors are ubiquitous, but the modulators are specific: P2X receptors in the sensory neurons are modulated by cannabinoids Pflugers Arch 2006 453 353 360 10.1007/s00424-006-0094-1 16741755

[B112] Hausmann R Rettinger J Gerevich Z Meis S Kassack MU Illes P Lambrecht G Schmalzing G The suramin analog 4,4',4",4"'-(carbonylbis(imino-5,1,3-benzenetriylbis (carbonylimino)))tetra-kis-benzenesulfonic acid (NF110) potently blocks P2X3 receptors: subtype selectivity is determined by location of sulfonic acid groups Mol Pharmacol 2006 69 2058 2067 10.1124/mol.106.022665 16551782

[B113] Jarvis MF Burgard EC McGaraughty S Honore P Lynch K Brennan TJ Brennan TJ Subieta A van Biesen T Cartmell J Bianchi B Niforatos W Kage K Yu H Mikusa J Wismer CT Zhu CZ Chu K Lee CH Stewart AO Polakowski J Cox BF Kowaluk E Williams M Sullivan J Faltynek C A-31 a novel potent and selective non-nucleotide antagonist of P2X_3 _and P2X_2/3 _receptors, reduces chronic inflammatory and neuropathic pain in the rat Proc Natl Acad Sci USA 7491 99 17179 17184 10.1073/pnas.252537299 PMC139289 12482951

[B114] Burnstock G Physiology and pathophysiology of purinergic neurotransmission Physiol Rev 2007 87 659 797 10.1152/physrev.00043.2006 17429044

[B115] Burnstock G Expanding field of purinergic signaling Drug Dev Res 2001 52 1 10 10.1002/ddr.1093

